# Off-pump vs. on-pump bypass surgery grafting in diabetic patients with three-vessel disease: a propensity score matching study

**DOI:** 10.3389/fcvm.2023.1249881

**Published:** 2023-11-30

**Authors:** Yu Song, Chen Wang, Chuanbin Tang, Xiaofan Huang, Dashuai Wang, Rui Li, Jingjing Luo, Yisilamujiang Tuerxun, Yuanming Li, Baoqing Liu, Long Wu, Xinling Du

**Affiliations:** ^1^ Department of Cardiovascular Surgery, Union Hospital, Tongji Medical College, Huazhong University of Science and Technology, Wuhan, China; ^2^ Department of Cardiovascular Surgery, The First Affiliated Hospital of Zhengzhou University, Zhengzhou, China; ^3^ Department of Cardiothoracic Surgery, The Second Affiliated Hospital of Xinjiang Medical University, Wulumuqi, China

**Keywords:** coronary artery bypass grafting, diabetes mellitus, off-pump, on-pump, propensity score matching

## Abstract

**Background:**

Controversy exists regarding the advantages and risks of off-pump vs. on-pump coronary artery bypass grafting (CABG) for patients with diabetes. We therefore compare the early clinical outcomes of off-pump vs. on-pump procedures for diabetic patients with three-vessel disease.

**Materials and methods:**

We conducted a retrospective analysis of clinical data obtained from 548 diabetic patients with three-vessel coronary artery disease who underwent isolated CABG between January 2016 and June 2020. To adjust the differences of baseline characteristics between the off-pump CABG (OPCAB) and on-pump CABG (ONCAB) groups, propensity score matching (PSM) was used. Following 1:1 matching, we selected 187 pairs of patients for further comparison of outcomes within the first 30 days after surgery.

**Results:**

The preoperative characteristics of the patients between the two groups were clinically comparable after PSM. The OPCAB group exhibited a significantly higher incidence of incomplete revascularization (27.3% vs. 14.4%; *P *= 0.002) compared with the ONCAB group. No differences were seen in mortality within 30 days between the matched groups (1.1% vs. 3.7%; *P *= 0.174). Notably, the OPCAB group had a lower risk of respiratory failure or infection (2.1% vs. 7.0%; *P *= 0.025), less postoperative stroke (1.1% vs. 4.8%; *P* = 0.032), and reduced postoperative ventilator assistance time (35.8 ± 33.7 vs. 50.9 ± 64.8; *P *= 0.005).

**Conclusion:**

OPCAB in diabetic patients with three-vessel disease is a safe procedure with reduced early stroke and respiratory complications and similar mortality rate, myocardial infarction, and renal failure requiring dialysis to conventional on-pump revascularization.

## 1. Introduction

Coronary artery disease (CAD) is virtually ubiquitous in diabetic patients and predicts a worse prognosis compared with those without diabetes (1). As incidence and prevalence have dramatically risen, so have the healthcare challenges of these individuals. The presence of diabetes mellitus (DM) in patients is associated with multivessel disease or severely stenosed vessels, which not only increases the risk of revascularization but also increases the risk of adverse outcomes after CABG or percutaneous coronary intervention (PCI) (2).

In diabetic patients, the benefit of CABG over PCI was indicated in many studies in terms of the risk of death, non-fatal stroke, non-fatal myocardial infarction, and need for repeat revascularization (3–5). Nevertheless, cardiopulmonary bypass (CPB) and cardioplegia-induced cardiac arrest have generally been performed during CABG (on-pump CABG, ONCAB), which is associated with systemic inflammatory response and complications such as respiratory complications and stroke (6). In order to avoid CPB and reduce postoperative complications, the technique of operating on a beating heart for CABG (off-pump CABG, OPCAB) was developed.

However, some studies have raised concerns about the potential high rate of incomplete revascularization and the effectiveness of revascularization with OPCAB, which is why the debate continues (7). Nevertheless, many studies have revealed that the results are at least as good as those of ONCAB and showed a particular benefit in some high-risk groups (8, 9).

The purpose of this study was to conduct an impartial evaluation of early prognosis between the two surgical methods used in treating diabetic patients with three-vessel disease by analyzing the clinical data obtained from our cardiac medical center.

## 2. Materials and methods

### 2.1. Patients

**Figure 1:**
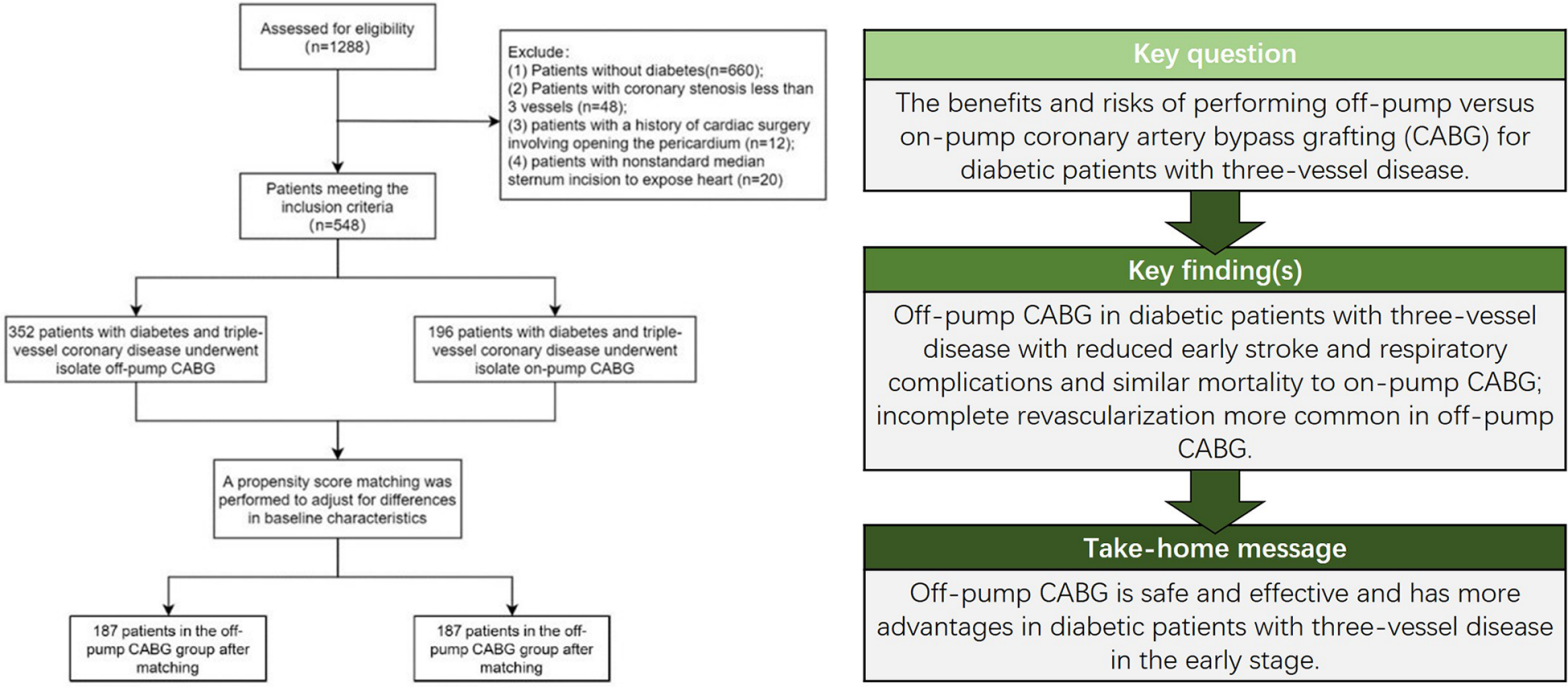
Key findings and flow chart of the study.

Between January 2011 and January 2021, a total of 1,288 patients underwent isolated CABG at our center. Patients without diabetes (*n *= 660), coronary stenosis in less than three vessels (*n *= 48), a history of cardiac surgery that involves opening the pericardium (*n *= 12), and non-standard median sternum incision to expose the heart (*n *= 20) were excluded. The final population comprised of 548 patients with DM, treated either through dietary interventions, oral antidiabetic medication, insulin therapy, or both. All patients received regular monitoring during hospitalization by a consulting physician from our diabetes center, and the immediate perioperative and postoperative management of their diabetic condition was overseen by diabetologists from the same center. Of these 548 patients, 352 received OPCAB, and 196 received ONCAB (Figure 1). All the operations were completed by experienced surgeons who had completed more than 500 on-pump CABGs and 250 off-pump CABGs. The Medical Ethics Committee of Tongji Medical College in Huazhong University of Science and Technology approved the ethics of this study (IORG No. IORG0003571), and a patient informed consent was waived.

### 2.2. Surgical technique

Both surgical methods utilized a standard incision in the middle of the sternum to expose the heart. In the OPCAB procedure, heparin was administered to achieve an active clotting time (ACT) of over 350 s and repeated as needed. A stabilizing agent (Octopus Tissue Stabilizer T2000, Medtronic, Minneapolis, MN, USA) was utilized to expose the target vessel. A shunt was regularly inserted, and a misting device with CO_2_ and water was employed to clear the surgical area. On the other hand, in the ONCAB procedure, the cardiopulmonary bypass is established by utilizing inflow cannulation of the aorta and outflow cannulation of the right atrium or outflow cannulation of the superior and inferior vena cava, along with intermittent antegrade cold blood cardioplegia for myocardial protection. In both approaches, grafts were obtained from the left internal mammary artery (LIMA), great saphenous vein, or radial artery. The conventional anastomosis involved connecting LIMA to the left anterior descending (LAD) artery and performing anastomosis of the great saphenous vein and/or anastomosis of the radial artery with other target vessels. All patients underwent regular ultrasound flow measurement of graft vessels. Apart from the differences in the two surgical procedures, anesthesia and patient management during hospitalization were similar.

### 2.3. Study variables

Preoperative patient data were collected, namely, age, sex, hypertension, hyperlipidemia, smoke, previous myocardial infarction, peripheral arterial disease (PAD), previous PCI, carotid artery stenosis, renal insufficiency, renal replacement therapy, stroke, chronic obstructive pulmonary disease (COPD), preoperative atrial fibrillation, left ventricle ejection fraction (LVEF), New York Heart Association (NYHA) class III or IV, urgent surgery, left ventricular end-diastolic diameter (LVEDD), and left main disease. Variables associated with revascularization included rate of LIMA use, number of distal anastomosis, and rate of incomplete revascularization. The primary early outcomes included mortality, non-fatal stroke, non-fatal myocardial infarction, and new cases requiring dialysis due to renal failure within 30 days. Other outcomes included new-onset atrial fibrillation occurrences, low cardiac output syndrome, new renal insufficiency, respiratory failure or infection, sternum Infection, and reoperation for bleeding. Clinical efficacy was also evaluated by postoperative LVEF, postoperative LVEDD, hospital stay time, postoperative intensive care unit (ICU) time, and ventilator assistance time.

### 2.4. Statistical analysis

In this study, patient data were obtained from the electronic medical record system of our center. Continuous variables with a normal distribution were expressed as mean ± standard deviation and analyzed using Student's *t*-test, while continuous variables with a non-normal distribution were expressed as interquartile range and analyzed using the Mann–Whitney *U*-test. Categorical variables were presented as percentages and analyzed using either the *χ*^2^ test or Fisher's exact test. To adjust for differences in baseline characteristics between the ONCAB and OPCAB groups, we performed 1:1 nearest neighbor propensity score matching (PSM) based on age, sex, smoking history, hypertension, hyperlipidemia, myocardial infarction, PCI, PAD, carotid artery stenosis, stroke, renal insufficiency, renal replacement therapy, COPD, atrial fibrillation, LVEF, LVEDD, NYHA class, urgent surgery, and left main artery stenosis. The balance of the two matched groups was evaluated by a standardized difference of the matched variables and illustrated by the love plot (Supplementary Figure S1). After matching, normally distributed continuous variables were analyzed using paired *t*-tests, while non-normally distributed continuous variables were analyzed using Wilcoxon tests. A *P*-value of <0.05 was considered statistically significant for all analyses conducted using IBM SPSS software (version 23, Armonk, NY, USA).

## 3. Results

### 3.1. Patient clinical characteristics

Between January 2016 and June 2020, a total of 548 patients diagnosed with diabetes mellitus who underwent isolated CABG were included in this study. Among them, 352 patients received OPCAB, and 196 patients received ONCAB.

Table 1 summarizes the demographics and preoperative variables before using the PSM analysis. In summary, both the OPCAB and ONCAB groups exhibited similar characteristics in terms of mean age, hypertension, smoke, hyperlipidemia, COPD, renal insufficiency, urgent operations, and history of PCI. However, the OPCAB group exhibited higher proportions of males (*P* < 0.001), carotid artery stenosis (*P *= 0.021), peripheral arterial disease (*P* = 0.001), NYHA class (>II, *P* = 0.001), and left main coronary artery stenosis (*P* = 0.013).

**Table 1:** Baseline characteristics of the patients before matching.

Characteristic	Off-pump CABG (*n* = 352)	On-pump CABG (*n* = 196)	*P*-value
Age, year	61.6 ± 9.2	60.3 ± 7.4	0.066
Male sex, *n* (%)	266 (75.6%)	120 (61.2%)	<0.001
Clinical history, *n* (%)			
Hypertension	311 (88.4%)	168 (85.7%)	0.372
Hyperlipidemia	246 (69.9%)	142 (72.4%)	0.527
Smoke	167 (47.4%)	81 (41.3%)	0.168
Myocardial infarction	140 (39.8%)	66 (33.2%)	0.125
PCI	50 (14.2%)	27 (13.8%)	0.890
Peripheral arterial disease	124 (35.2%)	42 (21.4%)	0.001
Carotid artery stenosis	48 (13.6%)	14 (7.1%)	0.021
Stroke	55 (15.6%)	23 (11.7%)	0.212
Renal insufficiency	20 (5.7%)	14 (7.1%)	0.497
Renal replacement therapy	3 (0.9%)	0 (0.0%)	0.556
COPD	48 (13.6%)	16 (8.2%)	0.056
Atrial fibrillation	5 (1.4%)	2 (1.0%)	1.000
LVEF, *n* (%)			
<35%	13 (3.7%)	3 (1.5%)	0.150
35%–49%	40 (11.4%)	15 (7.7%)	0.166
≥50%	299 (84.9%)	178 (90.8%)	0.050
Mean	59.2 ± 9.6	60.4 ± 7.7	0.096
LVEDD, cm	4.5 ± 0.5	4.4 ± 0.5	0.304
NYHA class >II, *n* (%)	40 (11.4%)	6 (3.1%)	0.001
Urgent surgery, *n* (%)	9 (2.6%)	1 (0.5%)	0.105
Left main coronary artery stenosis	77 (21.9%)	26 (13.3%)	0.013

Values are presented as mean ± standard deviation and *n* (%).

After matching, 187 pairs were selected in the two groups, and the preoperative baseline characteristics were comparable. Both OPCAB and ONCAB groups exhibited identical distributions across all variables (Table 2).

**Table 2:** Baseline characteristics of the patients after matching.

Characteristic	Off-pump CABG (*n* = 187)	On-pump CABG (*n* = 187)	*P*-value
Age, year	59.9 ± 9.1	60.4 ± 7.6	0.564
Male sex, *n* (%)	121 (64.7%)	118 (63.1%)	0.747
Clinical history, *n* (%)			
Hypertension	164 (87.7%)	164 (87.7%)	1.000
Hyperlipidemia	137 (73.3%)	134 (71.7%)	0.728
Smoke	83 (44.4%)	78 (41.7%)	0.319
Myocardial infarction	65 (34.8%)	65 (34.8%)	1.000
PCI	23 (12.3%)	25 (13.4%)	0.757
Peripheral arterial disease	46 (24.6%)	41 (21.9%)	0.541
Carotid artery stenosis	17 (9.1%)	14 (7.5%)	0.574
Stroke	24 (12.8%)	22 (11.8%)	0.753
Renal insufficiency	12 (6.4%)	13 (7.0%)	0.836
Renal replacement therapy	0 (0.0%)	0 (0.0%)	1.000
COPD	16 (8.6%)	16 (8.6%)	1.000
Atrial fibrillation	1 (0.5%)	2 (1.1%)	1.000
LVEF, *n* (%)			
<35%	5 (2.7%)	3 (1.6%)	0.479
35%–49%	16 (8.6%)	15 (8.0%)	0.851
≥50%	163 (87.2%)	169 (90.4%)	0.327
Mean	60.2 ± 9.8	60.4 ± 7.6	0.967
LVEDD, cm	4.8 ± 0.6	4.8 ± 0.5	0.899
NYHA class >II, *n* (%)	9 (4.8%)	6 (3.3%)	0.432
Urgent surgery, *n* (%)	1 (0.5%)	0 (0.0%)	1.000
Left main coronary artery stenosis	17 (9.1%)	22 (11.8%)	0.193

Values are presented as mean ± standard deviation and *n* (%).

### 3.2. Revascularization data

In the unadjusted analysis, the OPCAB group had a slightly lower number of distal anastomoses (3.4 ± 1.1 vs. 3.6 ± 0.8, *P *= 0.037) and a higher incidence of incomplete revascularization (25.3% vs. 14.8%, *P *= 0.004) compared with the ONCAB group. In addition, patients in the OPCAB group showed a higher use of LIMA (84.4% vs. 75.0%, *P *= 0.007) (Table 3). However, after the propensity matching was performed, the three variables mentioned above still had the same trend: less number of distal anastomoses (3.3 ± 1.1 vs. 3.6 ± 0.8, *P *= 0.003), higher rate of incomplete revascularization (27.3% vs. 14.4%, *P *= 0.002), and more use of LIMA (84.5% vs. 74.9%, *P* = 0.021) (Table 4).

**Table 3:** Operative characteristics and early outcome of the patients before matching.

Characteristics	Off-pump CABG (*n* = 352)	On-pump CABG (*n* = 196)	*P*-value
Operative characteristics			
No. of distal anastomosis, mean	3.4 ± 1.1	3.6 ± 0.8	0.037
LIMA use, *n* (%)	297 (84.4%)	147 (75.0%)	0.007
Incomplete revascularization, *n* (%)	89 (25.3%)	29 (14.8%)	0.004
Ventilator assistance time, h, mean	42.3 ± 82.4	49.9 ± 63.5	0.333
Postoperative ICU stay, days, mean	4.5 ± 4.5	4.7 ± 3.9	0.545
Hospital stay time, days, mean	30.6 ± 11.0	29.5 ± 10.1	0.254
Mean of postoperative LVEF, %	57.7 ± 8.0	58.4 ± 6.9	0.309
Mean of postoperative LVEDD, cm	4.5 ± 0.5	4.4 ± 0.5	0.220
Early outcome			
Primary outcome, *n* (%)
Death	16 (4.5%)	7 (3.6%)	0.586
Myocardial infarction	10 (2.8%)	3 (1.5%)	0.396
Stroke	8 (2.3%)	9 (4.6%)	0.133
Renal failure requiring dialysis	18 (5.1%)	5 (2.6%)	0.152
Other outcome, *n* (%)
Low cardiac output syndrome	34 (9.7%)	14 (7.1%)	0.318
New-onset atrial fibrillation	8 (2.3%)	2 (1.0%)	0.507
Respiratory failure or infection	20 (5.7%)	13 (6.6%)	0.654
Renal insufficiency	68 (19.3%)	39 (19.9%)	0.870
Sternum infection	3 (0.9%)	4 (2.0%)	0.255
Reoperation for bleeding	7 (2.0%)	4 (2.0%)	1.000

Values are presented as mean ± standard deviation and *n* (%).

**Table 4:** Operative characteristics and early outcome of the patients after matching.

Characteristics	Off-pump CABG (*n* = 187)	On-pump CABG (*n* = 187)	*P*-value
Operative characteristics
No. of distal anastomosis, mean	3.3 ± 1.1	3.6 ± 0.8	0.003
LIMA use, *n* (%)	158 (84.5%)	140 (74.9%)	0.021
Incomplete revascularization, *n* (%)	51 (27.3%)	27 (14.4%)	0.002
Ventilator assistance time, h, mean	35.8 ± 33.7	50.9 ± 64.8	0.005
Postoperative ICU stay, days, mean	4.1 ± 3.1	4.8 ± 4.0	0.065
Hospital stay time, days, mean	30.7 ± 11.3	29.5 ± 10.0	0.273
Mean of postoperative LVEF, %	58.3 ± 8.1	58.2 ± 6.9	0.950
Mean of postoperative LVEDD, cm	4.4 ± 0.5	4.4 ± 0.5	0.936
Early outcome			
Primary outcome, *n* (%)
Death	2 (1.1%)	7 (3.7%)	0.174
Myocardial infarction	0 (0.0%)	3 (1.6%)	0.248
Stroke	2 (1.1%)	9 (4.8%)	0.032
Renal failure requiring dialysis	5 (2.7%)	4 (2.1%)	1.000
Other outcome, *n* (%)
Low cardiac output syndrome	13 (7.0%)	14 (7.5%)	0.842
New-onset atrial fibrillation	3 (1.6%)	2 (1.1%)	1.000
Respiratory failure or infection	4 (2.1%)	13 (7.0%)	0.025
Renal insufficiency	30 (16.0%)	38 (20.3%)	0.283
Sternum infection	3 (1.6%)	3 (1.6%)	1.000
Reoperation for bleeding	3 (1.6%)	4 (2.1%)	1.000

Values are presented as mean ± standard deviation and *n* (%).

### 3.3. Early primary outcome

In unadjusted studies, no significant differences were observed between the two groups in terms of the early postoperative primary outcome, such as death (*P* = 0.586), stroke (*P* = 0.396), non-fatal myocardial infarction (*P* = 0.133), and new renal failure requiring dialysis (*P* = 0.152) (Table 3).

After the propensity matching analysis, no statistically significant differences were found for death (*P* = 0.174), new renal failure requiring dialysis (*P* = 1.000), and non-fatal myocardial infarction (*P* = 0.248) between the two groups. Nevertheless, it is worth noting that OPCAB demonstrated a favorable effect in reducing postoperative stroke incidence (1.1% vs. 4.8%, *P* = 0.032).

### 3.4. Early other outcome

In the initial unadjusted studies, the patients in the OPCAB group and the ONCAB group did not display any differences in the incidence of early postoperative other outcome such as low cardiac output syndrome (*P* = 0.318), new-onset atrial fibrillation (*P* = 0.507), respiratory failure or infection (*P* = 0.654), new renal insufficiency (*P* = 0.870), reoperation for bleeding (*P* = 1.000), and sternum infection (*P* = 0.255).

After the propensity matching was performed, it was found that OPCAB had lower incidence of respiratory failure or infection (2.1% vs. 7.0%, *P* = 0.025) and reduced postoperative ventilator assistance time (35.8 ± 33.7 vs. 50.9 ± 64.8, *P* = 0.005). As with unmatched results, the rest of the early other outcome was similar in both groups.

## 4. Discussion

In the present study, it was observed that OPCAB had a lower occurrence of postoperative stroke, respiratory failure or infection, and a shorter duration of postoperative ventilator assistance in diabetic patients with three-vessel coronary artery disease who underwent CABG. Meanwhile, no significant differences were found in the rate of death, non-fatal myocardial infarction, or non-fatal new renal failure requiring dialysis within 30 days. These findings suggest that compared with ONCAB, OPCAB, performed by experienced surgeons specializing in CABG with high surgical volumes, may help reduce early postoperative complications in diabetic patients with three-vessel disease.

The controversy over OPCAB and ONCAB is still ongoing, with a subgroup analysis proving to be of greater value (7, 10). The consensus on this question is that OPCAB may be linked to increased risks of long-term adverse events; however, it could potentially offer advantages in terms of reducing early procedural risks compared with ONCAB, particularly among high-risk individuals. Therefore, patients with diabetes who undergo CABG may benefit more from OPCAB because they are often accompanied by more diffuse and severe vascular disease and worse tolerance to surgery. Emmert et al. (11) compared short-term outcomes in 1,015 diabetic patients with three-vessel disease who underwent coronary revascularization. Of these patients, those who underwent OPCAB (540) had lower mortality rates and better postoperative outcomes, such as renal failure, pleural effusions, respiratory failure, and rethoracotomy for bleeding compared with those who underwent ONCAB (475), confirming the advantage of OPCAB on short-term outcomes for this patient population. The larger randomized trial CORONARY (12) showed comparable rates of the composite outcome comprising death, stroke, myocardial infarction, renal failure, or repeat revascularization after 5 years of follow-up between patients who underwent OPCAB and those who underwent ONCAB. However, when analyzing individuals with diabetes, a noteworthy increase in the incidence of stroke was observed among those who received ONCAB. Renner et al. (13) compared 355 OPCAB and 502 ONCAB procedures and concluded that off-pump surgery may be more beneficial for diabetic patients due to a lower risk of mortality within the first 30 days after surgery as well as in the mid-term. Results coincide with a lower rate of postoperative neurologic complications, less renal replacement therapy, and a shorter ventilator assistance time in diabetic patients who undergo OPCAB. A meta-analysis (14) showed that OPCAB imparts some survival benefit to patients with higher risk such as those undergoing redo CABG, diabetics, and the elderly who may gain the most benefit. However, not all studies have similar results. A subgroup analysis of diabetic patients in the ROOBY trial (15) reported that OPCAB yielded no advantage over ONCAB for diabetic patients including less complete revascularization index, a slight increase in 30-day adverse events, and a slightly higher 30-day mortality rate. However, surgeons who were relatively inexperienced in performing off-pump surgery were widely questioned in the ROOBY trial, undermining the reliability of the conclusions. In the present study, surgeons with extensive experience in performing OPCAB narrowed the effect of this factor. In conjunction with our findings, the potential benefit of OPCAB performed in treating diabetic patients with three-vessel disease may lie in mitigating or reducing non-fatal complications.

Similar to other studies (16–18), our study also showed that OPCAB surgery had a significant advantage over ONCAB in terms of cerebrovascular protection. We found a more than 4-fold reduction in the incidence of cerebrovascular complications following OPCAB compared with ONCAB among diabetic patients. With the help of transcranial Doppler ultrasonography, Bowles et al. (19) reported a significantly higher occurrence of cerebral microemboli in patients who underwent ONCAB compared with those who underwent OPCAB (1,766 vs. 27, *P* = 0.003). Furthermore, multivariate analysis identified that the type of surgery (OPCAB vs. ONCAB) was strongly associated with the number of microemboli detected (*P* = 0.002), which may increase the risk of adverse neurological events. In a meta-analysis involving 13 studies and a total of 37,720 patients (20), OPCAB was associated with a significant decrease in the risk of stroke within 30 days. Specifically, the reduction was observed to be 78% when compared with traditional ONCAB using aortic cross-clamping, 66% when compared with off-pump using partial clamping, and 52% when compared with off-pump utilizing the clampless HeartString device. These data suggest that the reduced manipulation of the aorta is most likely associated with a decreased incidence of stroke. In relation to this matter, it is necessary to consider three types of aortic manipulation, namely, (1) insertion of the arterial CPB cannula as well as the high speed jet from the cannula, (2) total aortic clamping for cardioplegic arrest, and (3) partial clamping for suturing the proximal anastomoses (21). In our study, the higher rate of incomplete revascularization in the OPCAB group suggested a reduced partial clamping, which may potentially contribute to the reduced incidence of short-term postoperative stroke. Therefore, we further examined the incidence of stroke in both complete and incomplete revascularization groups before (3.3% vs. 2.5%, *P* = 1.000) and after matching (0.0% vs. 3.8%, *P* = 0.130), and the results showed no statistically significant difference. The findings suggest, in part, that the observed decrease in stroke occurrence within the OPCAB group in our study cannot be solely attributed to a lower incidence of side-wall aortic clamping caused by incomplete revascularization. Therefore, a more reasonable explanation may be that the potential benefits of OPCAB in stroke are likely to be influenced by the combination of all three types of aortic manipulations. In short, by avoiding the use of a bypass circuit or clamping the aorta, it is effective to decrease the release of aortic atherosclerotic and calcified debris, as well as the microgaseous and microparticulate emboli, thereby reducing the postoperative neurologic complications.

Pulmonary function is significantly impacted following heart surgery, and the respiratory failure is a prevalent and severe complication following CABG that can have great adverse effects on both recovery and survival. In our study, OPCAB was associated with a higher potential for reducing postoperative respiratory compliance and the time of ventilator assistance time. Compared with OPCAB, the use of CPB and cardiac arrest during ONCAB is considered to result in more severe pulmonary dysfunction. Various studies confirmed that CPB, an inherently unnatural process, magnifies the reaction of inflammatory response (22, 23). According to the findings of Staton et al. (22), patients who underwent CABG with CPB actually had worse gas exchange ability immediately after surgery and a delay in extubation. Interestingly, these patients did not exhibit signs of pulmonary edema and showed only a slight decrease in lung compliance. The researchers hypothesized that these observations may lie in some aspect of CPB, such as the release of inflammatory mediators or potential impairment in surfactant replenishment due to inadequate ventilation cycling within the lungs. These evidences indicate that OPCAB is a viable alternative for high-risk patients with respiratory failure who require CABG.

The incidence of incomplete revascularization in our study was found to be higher in OPCAB compared with ONCAB no matter before (25.3% vs. 14.8%, *P* = 0.004) or after (27.3% vs. 14.4%, *P* = 0.002) matching. This difference is related to the definition of incomplete revascularization in this study; any coronary artery mentioned in coronary angiography of the patient with significant lesions (>50%), including the left anterior descending, left circumflex, right coronary artery, or even the larger branches of these vessels that have not be revascularized is defined as incomplete revascularization, which is basically consistent with and even more strict than the anatomical definition used in previous studies (14). In fact, our previous report, which involved diabetic patients in this study, showed a lower rate of incomplete revascularization in OPCAB (12.4%) when compared with various large-scale prospective multicenter clinical trials comparing ONCAB and OPCAB, such as the ROOBY trial (17.8%) (24) and GOPCABE trial (34%) (25). On the other hand, patients in the OPCAB group of the present study had higher surgical risk with higher proportions of peripheral vascular disease (35.2% vs. 21.4%, *P* = 0.001), carotid artery stenosis (13.6% vs. 7.1%, *P* = 0.021), COPD prevalence (13.6% vs. 8.2%, *P* = 0.056), lower LVEF (LVEF ≥ 50% 84.9% vs. 90.8%, *P* = 0.050), and higher NYHA class (>Ⅱ, 11.4% vs. 3.1%, *P* = 0.001). It is noteworthy that in patients with a higher-risk profile, the off-pump technique was primarily selected to reduce patient trauma by combining it with target revascularization. The culprit lesion, typically the LAD, was identified and treated, while other diseased vessels were left untreated to minimize the procedure (26). Consistent with this view, in this study, the left main coronary artery stenosis rate was higher in the OPCAB group (21.9% vs. 13.3%, *P* = 0.013) before matching. Of course, many studies (27–29) showed that the higher rate of incomplete revascularization in OPCAB led to higher rates of subsequent revascularization, which resulted in the inferior long-term outcome than ONCAB. Further, when this factor was analyzed in patients with diabetes, the difference was even more significant because it is well known that vascular lesions, especially small vessels, are more common in diabetic patients. Evidence suggesting that the presence of small-vessel disease or an increase in coronary microvascular resistance may contribute to persistent angina symptoms and morbidity, regardless of the extent of revascularization performed on the epicardial coronary arteries, which highlights the need for alternative therapeutic approaches was demonstrated (27, 30). Hence, in our study, some arteries were not selected as target vessels before operation. Meanwhile, with the development of new assistive technologies including suction-based apical cardiac positioning devices, intracoronary shunts, and new strategies proposed such as “functional complete revascularization,” the advantages of anatomical complete revascularization faded out (31). However, we would like to emphasize that surgeons should not be misled by the results into considering that incomplete revascularization is reasonable in OPCAB. Because with the help of cardioplegia that provides a longer arrest period (32), CABG can be performed at a higher quality in more heart centers. According to the findings of this study, we conclude that in medical centers where techniques are highly advanced and operators possess extensive experience, the OPCAB approach does not come at the cost of less complete revascularization in the early postoperative period.

This propensity-matched study has a number of limitations. First, due to the retrospective nature of the study, some confounding factors cannot be avoided. Second, since it was conducted at a single center, the generalizability of our findings needs further discussion. In addition, we were unable to obtain follow-up data for assessing surgical efficacy. In addition, the study lacked the information about the number of patients receiving diet, oral, or insulin treatment in each group, which is also important for evaluating the efficacy of surgery. Finally, while propensity matching can address selection bias, potential confounding, and covariate imbalance, it does have drawbacks as some individuals end up not matching and thus get excluded from the analysis resulting in a loss of both precision and generalizability.

## 5. Conclusion

In summary, our data demonstrate that in patients with diabetes, OPCAB effectively reduces the incidence of postoperative stroke and respiratory complications and provides survival comparable to ONCAB within 30 days. However, the high incidence of incomplete revascularization should not be ignored, and long-term follow-up may further refine our evaluation of off-pump coronary revascularization. For patients with diabetes who were preoperatively evaluated for a high risk of stroke and respiratory failure, OPCAB can provide a significant benefit.

## Data Availability

The raw data supporting the conclusions of this article will be made available by the authors, without undue reservation.
