# Supplementary material for: Off-pump vs. on-pump bypass surgery grafting in diabetic patients with three-vessel disease: a propensity score matching study
Source: Front Cardiovasc Med. 2023 Nov 30;10:1249881. doi: 10.3389/fcvm.2023.1249881 (PMC10719841; doi:10.3389/fcvm.2023.1249881)
Supplement: Supplementary file 1 [file Datasheet1.pdf]

Off-Pump Versus On-Pump Bypass Surgery Grafting in diabetic patients with three-vessel disease: A Propensity Score Matching Study

Yu Song[1,†], Chen Wang[1,†], Chuanbin Tang[1,†], Xiaofan Huang[1], Dashuai Wang[2], Rui Li[1], Jingjing Luo[1], Yisilamujiang Tuerxun[3], Yuanming Li[3], Baoqing Liu[1,*], Long Wu[1,*], and Xinling Du[1,*]

[1] Department of Cardiovascular Surgery, Union Hospital, Tongji Medical College, Huazhong University of Science and Technology, Wuhan, China.

[2] Department of Cardiovascular Surgery, The First Affiliated Hospital of Zhengzhou University, Zhengzhou, China.

[3] Department of Cardiothoracic Surgery, the Second Affiliated Hospital of Xinjiang Medical University, Wulumuqi, China.

*** Correspondence:**
liubq511@sina.com (B.L.); wulong@hust.edu.cn (L.W.); xinlingdu@hust.edu.cn (X.D.)

† These authors contributed equally to this work.

1 Supplementary Data

The variables are defined as follows

1.1 Hypertension was defined as systolic blood pressure ⩾ 140 mmHg and/or diastolic blood pressure ⩾ 90 mmHg were measured at least twice in the resting state or needed to take blood pressure lowering drugs to maintain blood pressure in the normal range, and secondary hypertension was excluded [1].

1.2. Hyperlipidemia was defined as fasting venous blood biochemical test indicators meet at least one of the following [2]: triglyceride (TC) ⩾ 5.2mmol/L (200mg/dl) or low-density lipoprotein (LDL-C) ⩾ 3.4mmol/L (130mg/dl) or high-density lipoprotein (HDL-C) <1.0 mmol/L (40mg/dl).

1.3. Diabetes was defined as typical diabetic symptoms (polyuria, polydipsia, and weight loss) combined with any time blood glucose ⩾11.1 mmol/L (200 mg/dl) or fasting blood glucose ⩾7.0 mmol/L (126 mg/dl) or oral glucose tolerance test (OGTT) ⩾ 11.1mmol/L (200mg/dl) [3].

1.4. COPD

Patients with chronic cough, expectoration, worsening dyspnea and forced expiratory volume in one second occupied force vital capacity percentage (FEV1/FVC) less than 70%.

1.5. Carotid artery and renal artery stenosis

The vascular ultrasonography or vascular CTA examination reported the stenosis area of the artery cross-sectional area exceeds 50% as stenosis.

1.6. Atrial fibrillation

The diagnosis of atrial fibrillation is based on the electrocardiogram and physical examination.

1.7. Coronary artery lesions.

The number of coronary artery lesions and the location of coronary artery lesions were determined according to the results of coronary angiography within 6 months. Coronary arteries with more than 50% stenosis are considered diseased vessels and isolated left main disease was identified as 2-vessel disease. Left anterior descending, left circumflex, and right coronary artery branch lesions were also included in these three artery lesions.

1.8. Preoperative left ventricular ejection fraction (LVEF) and left ventricular end-diastolic diameter (LVEDD)

The data were obtained from outpatient or preoperative echocardiography within 30 days before the operation.

1.9. Postoperative LVEF and LVEDD

The data were obtained according to the results of the postoperative in-hospital echocardiography.

1.10. Emergency surgery

CABG was performed within 24 hours of hospitalization or emergency CABG due to acute myocardial infarction and cardiac catheterization complications.

1.11. Postoperative ventilator assistance and ICU time were calculated according to the nursing record sheet and doctor order sheet.

1.12. Incomplete revascularization is defined as any coronary artery mentioned in patient’s coronary angiography with significant lesions (>50%) that was not revascularized.

1.13. Stroke

A new acute focal neurological deficit thought to be of vascular origin with signs or symptoms lasting longer than 24 hours. Strokes were confirmed by a neurologist based on clinical manifestations combined with brain CT and (or) magnetic resonance examination results.

1.14. Postoperative myocardial infarction

The actual value of myocardial enzymes in postoperative biochemical detection is higher than 10 times the 99th percentile of the normal distribution of the normal reference value and accompanied by one of the following conditions [4]: (1) The ECG has new left bundle branch block; (2) New graft occlusion or new native coronary artery occlusion on angiography; (3) Imaging evidence of new loss of viable myocardium or new regional wall motion abnormalities.

1.15. Postoperative low cardiac output syndrome (LCOS)

Postoperative LCOS is diagnosed when at least one of the following conditions [5]: (1) The patient needs primary care in the operating room or intensive care unit due to hemodynamic instability. Intra-arterial balloon pump (IABP) to end cardiopulmonary bypass or intensive care unit; (2) patients need two or more vasopressors (dopamine, dobutamine, epinephrine, norepinephrine, isoproterenol) to maintain systolic blood pressure greater than 90 mmHg and cardiac output greater than 2.2 L·min·m2.

1.16. New-onset atrial fibrillation

The diagnosis of postoperative new-onset atrial fibrillation was based on electrocardiogram and physical examination.

1.17. Renal insufficiency

Renal insufficiency refers to glomerular filtration rate < 90ml/min or detected serum creatinine value higher than 133umol/L.

1.18. Postoperative respiratory failure

This included respiratory failure (i.e., requiring re-intubation and mechanical ventilation) and severe respiratory infection.

1.19. Repeat thoracotomy due to hemorrhage

The postoperative drainage volume exceeds 200ml per hour and lasts for more than 3 hours, blood pressure and hemoglobin levels continue to decline, and surgical intervention is required to open the chest to stop bleeding.

1.20. Sternal infection

Diagnosis of sternal infection is based on physical examination, imaging, and bacterial culture

Reference

1. Flack JM, Adekola B. Blood pressure and the new ACC/AHA hypertension guidelines. Trends Cardiovasc Med (2020) 30:160-4. 10.1016/j.tcm.2019.05.003

2. Berglund L, Brunzell JD, Goldberg AC, Goldberg IJ, Sacks F, Murad MH, et al. Evaluation and treatment of hypertriglyceridemia: an Endocrine Society clinical practice guideline. J Clin Endocrinol Metab (2012) 97:2969-89. 10.1210/jc.2011-3213

3. Li Y, Teng D, Shi X, Qin G, Qin Y, Quan H, et al. Prevalence of diabetes recorded in mainland China using 2018 diagnostic criteria from the American Diabetes Association: national cross sectional study. BMJ (2020) 369:m997. 10.1136/bmj.m997

4. Thygesen K, Alpert JS, Jaffe AS, Chaitman BR, Bax JJ, Morrow DA, et al. Fourth Universal Definition of Myocardial Infarction (2018). J Am Coll Cardiol (2018) 72:2231-64. 10.1016/j.jacc.2018.08.1038

5. Algarni KD, Maganti M, Yau TM. Predictors of low cardiac output syndrome after isolated coronary artery bypass surgery: trends over 20 years. Ann Thorac Surg (2011) 92:1678-84. 10.1016/j.athoracsur.2011.06.017

2 Supplementary Figures and Tables

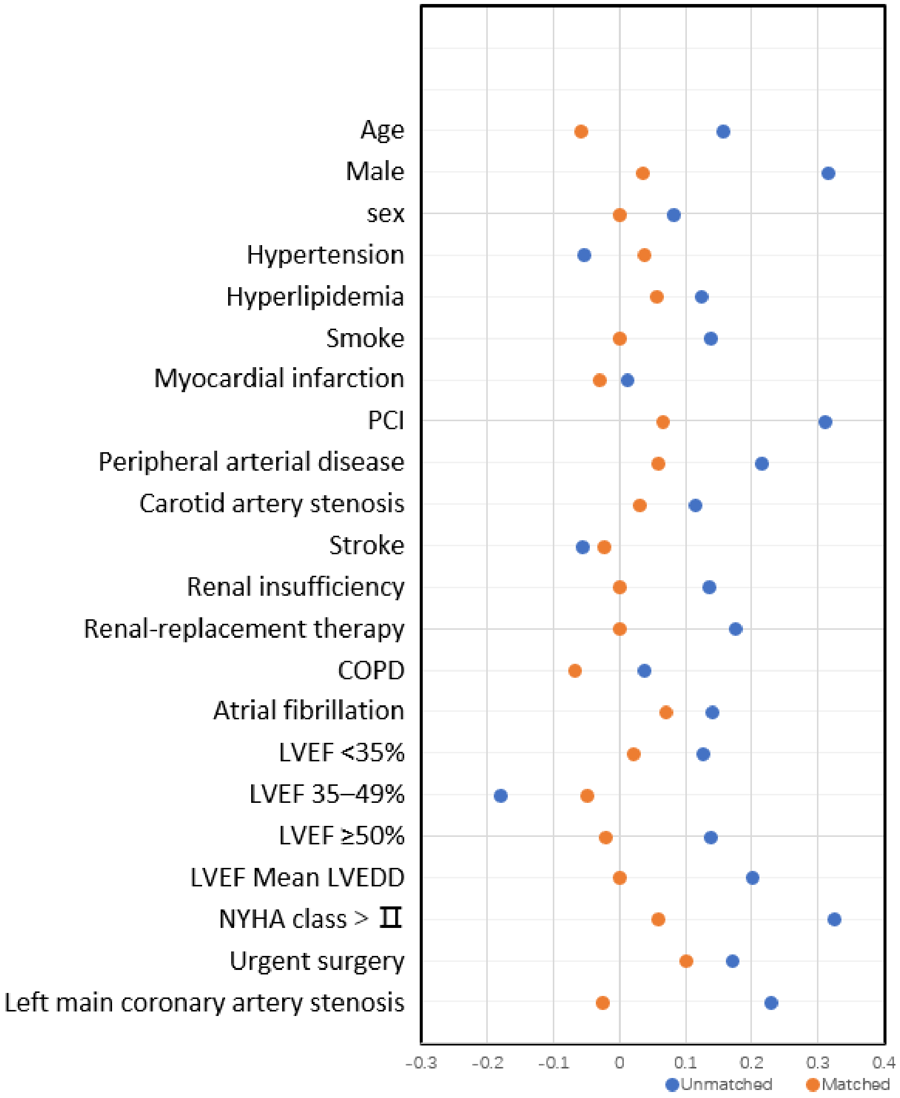

Supplementary Figure 1. The love plot to illustrate the covariates balance of the two matched groups.
